# Estimating the Burden of Paratyphoid A in Asia and Africa

**DOI:** 10.1371/journal.pntd.0002925

**Published:** 2014-06-05

**Authors:** Michael B. Arndt, Emily M. Mosites, Mu Tian, Mohammad H. Forouzanfar, Ali H. Mokhdad, Margaret Meller, Rion L. Ochiai, Judd L. Walson

**Affiliations:** 1 Department of Epidemiology, University of Washington, Seattle, Washington, United States of America; 2 Department of Health Services, University of Washington, Seattle, Washington, United States of America; 3 Institute for Health Metrics and Evaluation, Seattle, Washington, United States of America; 4 Department of Global Health, University of Washington, Seattle, Washington, United States of America; 5 The Bill and Melinda Gates Foundation, Seattle, Washington, United States of America; 6 Sanofi Pasteur, Lyon, France; 7 Department of Medicine, University of Washington, Seattle, Washington, United States of America; 8 Department of Pediatrics, University of Washington, Seattle, Washington, United States of America; Oxford University Clinical Research Unit, Viet Nam

## Abstract

Despite the increasing availability of typhoid vaccine in many regions, global estimates of mortality attributable to enteric fever appear stable. While both *Salmonella enterica* serovar Typhi (*S.* Typhi) and serovar Paratyphi (*S.* Paratyphi) cause enteric fever, limited data exist estimating the burden of *S.* Paratyphi, particularly in Asia and Africa.

We performed a systematic review of both English and Chinese-language databases to estimate the regional burden of paratyphoid within Africa and Asia. Distinct from previous reviews of the topic, we have presented two separate measures of burden; both incidence and proportion of enteric fever attributable to paratyphoid. Included articles reported laboratory-confirmed *Salmonella* serovar classification, provided clear methods on sampling strategy, defined the age range of participants, and specified the time period of the study.

A total of 64 full-text articles satisfied inclusion criteria and were included in the qualitative synthesis. Paratyphoid A was commonly identified as a cause of enteric fever throughout Asia. The highest incidence estimates in Asia came from China; four studies estimated incidence rates of over 150 cases/100,000 person-years. Paratyphoid A burden estimates from Africa were extremely limited and with the exception of Nigeria, few population or hospital-based studies from Africa reported significant Paratyphoid A burden.

While significant gaps exist in the existing population-level estimates of paratyphoid burden in Asia and Africa, available data suggest that paratyphoid A is a significant cause of enteric fever in Asia. The high variability in documented incidence and proportion estimates of paratyphoid suggest considerable geospatial variability in the burden of paratyphoid fever. Additional efforts to monitor enteric fever at the population level will be necessary in order to accurately quantify the public health threat posed by *S.* Paratyphi A, and to improve the prevention and treatment of enteric fever.

## Author Summary

Enteric fever due to *Salmonella enterica* serovar Typhi (*S.* Typhi) and serovar Paratyphi (*S.* Paratyphi A, B, C) remains a global public health concern. While numerous studies have estimated the levels or burden of *S.* Typhi, there are only limited data estimating the burden of *S.* Paratyphi A, particularly in Asia and Africa. We reviewed both English and Chinese-language databases for estimates of the regional burden within Africa and Asia, including new paratyphoid A cases/year and proportion of enteric fever cases attributable to paratyphoid A. *S.* Paratyphi A appears to constitute a significant proportion of all cases of enteric fever in Asia, though population level estimates are limited, especially in Africa. There was high variability in reported paratyphoid A burden estimates, which suggests considerable geospatial variability in the burden of paratyphoid fever. Improved efforts to monitor enteric fever at the population level are warranted in order to correctly measure the public health threat posed by *S.* Paratyphi A and to determine the potential need for *S.* Paratyphi specific prevention and treatment interventions.

## Introduction

The global burden of enteric fever is significant, with millions of cases reported each year [1]. In 2010 enteric fever was responsible for an estimated 190,000 deaths and more than 12.2 million disability adjusted life years (DALYs) [2], [3]. Despite increased availability of typhoid vaccine, the age-standardized mortality rate from enteric fever has remained around 3 per 100,000 during the past 20 years globally, and higher in developing countries [2]. While both *Salmonella enterica* serovar Typhi (*S.* Typhi) and serovar Paratyphi (*S.* Paratyphi A, B, and C) are recognized as causes of enteric fever, limited data exist on the burden of *S.* Paratyphi globally, particularly in Asia and Africa where the majority of reported enteric fever morbidity and mortality occurs [1], [2], [4], [5]. Reporting of paratyphoid B and C is infrequent, and studies which have provided separate estimates for *S.* Paratyphi A and *S.* Typhi suggest that *S.* Paratyphi A may account for a considerable proportion of enteric fever in Asian countries [5]. However, most studies of enteric fever in these regions do not distinguish between *S.* Paratyphi A and *S.* Typhi and the true burden of *S.* Paratyphi A as a cause of enteric fever is not well documented.

We performed a systematic review of both English and Chinese-language databases to estimate the regional burden of paratyphoid A within Africa and Asia. Distinct from previous reviews of the topic, we have presented two separate measures of burden; both incidence and proportion of enteric fever attributable to paratyphoid A. Available data are limited but suggest that *S.* Paratyphi A accounts for a substantial proportion of enteric fever cases in some areas, although there appears to be significant geographic variability in incidence. The lack of systematic surveillance for *S.* Paratyphi A makes reliable estimation of burden difficult. Given the reported incidence and proportion of enteric fever cases attributable to *S.* Paratyphi A, increased attention to this disease is warranted, particularly as *S.* Typhi vaccine coverage increases.

## Methods

### Search strategy and selection criteria

References for this review were identified through searches of both English and Chinese-language databases for published literature on paratyphoid burden, in accordance with the PRISMA guidelines. We searched PubMed, Embase, and Web of Science for the English-language literature review and the China National Knowledge Infrastructure (CNKI) database for the Chinese literature review. The CNKI database includes articles from over 7,500 journals and is considered the most comprehensive collection of research articles from China. Two authors (MA and EM) conducted the search of the English-language databases and a third author (MT) conducted the search of the CNKI database.

### Eligibility criteria

For inclusion in the database review, we restricted to articles which were conducted in Africa or Asia, reported the laboratory-confirmed *Salmonella* serovar classification, provided clear methods on sampling strategy, defined the age range of participants, and specified the time period of the study. Laboratory confirmation of paratyphoid required detection of *S. enterica* by blood or bone-marrow culture, followed by serological and/or biochemical methods for species identification. We chose to capture results for paratyphoid A because the reporting of paratyphoid B and C were relatively infrequent.

Appropriate study designs for assessing *S.* Paratyphi A disease burden included population-based surveillance studies, cohort studies, and hospital or laboratory-based studies (although Chinese-language studies were restricted to population-based studies). Studies were excluded if no distinction was made between paratyphoid and typhoid, or where paratyphoid burden was calculated as a constant proportion of total enteric fever cases. Where two articles reported information from the same study, the later publication was excluded. As we felt that program and policy implications related to enteric fever control should depend on the current burden, we restricted our search to papers published from January 2005 to May 2013. No language restrictions were prescribed.

### Search

The PubMed search terms included: Paratyphi[All Fields] OR (“paratyphoid fever”[MeSH Terms] OR (“paratyphoid”[All Fields] AND “fever”[All Fields]) OR “paratyphoid fever”[All Fields] OR “paratyphoid”[All Fields]) AND (“incidence”[All Fields] OR “prevalence”[All Fields] OR “burden”[All Fields] OR “estimates”[All Fields]).

The Web of Science search terms included: TS = ((“salmonella Paratyphi” OR paratyphoid OR “enteric fever”)) AND TS = ((incidence OR burden OR prevalence)) AND TS = ((Asia OR Africa)).

The Embase search terms included: ‘salmonella Paratyphi’/de OR ‘paratyphoid’/de OR ‘enteric fever’/de AND (‘incidence’/de OR burden OR ‘prevalence’/de) AND (‘Asia’/de OR ‘Africa’/de) AND [Embase]/lim.

The CNKI search terms included: “Paratyphi and incidence” or “Paratyphi and prevalence” or “Paratyphi and burden” (发表时间 between(1990-01-01,2013-01-18) 并且 (摘要 = 副伤寒 并且 摘要 = 发病率 或者 摘要 = 副伤寒 并且 摘要 = 患病率 或者 摘要 = 副伤寒 并且 摘要 = 负担) (精确匹配).

The term “enteric fever” was added to Embase and Web of Science search terms to ensure sufficient breadth of search returns. An initial search was completed in November 2012. The search was last run on May 2, 2013 to ensure that no articles had been missed in the intervening period.

### Study selection and data collection process

Articles were initially screened for relevance based on the title and abstract. Those articles with the potential to fit the eligibility criteria were fully reviewed. We extracted data from each study into a table, including global region, study location, period of data collection, setting, sample or population size, study design, patient age and health status, diagnostic technique, species identification technique, number of *S.* Paratyphi A cases, number of *S.* Typhi cases, *S.* Paratyphi A proportion, *S.* Paratyphi A incidence, *S.* Typhi incidence, quality score, and bibliographic information. For the articles that did not enumerate the population used to estimate *S.* Paratyphi A incidence, the population catchment area was sourced from census data.

For population-based study designs, we extracted estimates of the incidence of *S.* Paratyphi A (per 100,000 person-years). For hospital and laboratory-based studies, the burden of *S.* Paratyphi A was only available as the proportion of enteric fever cases that were *S.* Paratyphi A (compared to typhoid). However, these proportion estimates are not considered representative of the population burden.

### Risk of bias in individual studies

**Figure 1:**
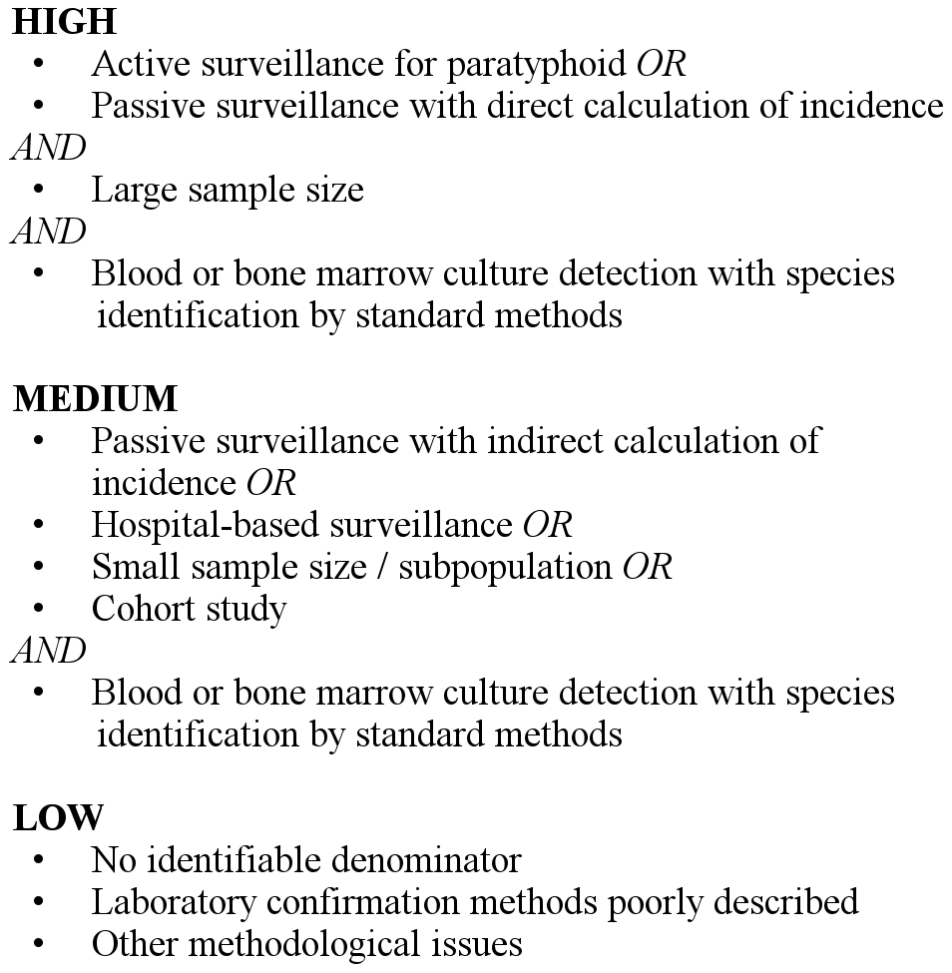
Quality score criteria.

Quality assessments were independently performed by one of two reviewers using a pre-defined quality score, which ranked study quality based on the criteria in Figure 1. Where necessary, reviewers discussed and jointly scored articles to assign a quality score based on predefined criteria.

### Synthesis of results

The studies of paratyphoid in Asia are presented by region (South Asia, Southeast Asia, and East Asia) and country. Due to the limited data from Africa, we summarized estimates of paratyphoid A in Africa by country, but not region. Within each geographic region, studies are presented by study design to separate those which provided incidence and those which provided the proportion of enteric fever cases which were due to paratyphoid A.

## Results

**Figure 2:**
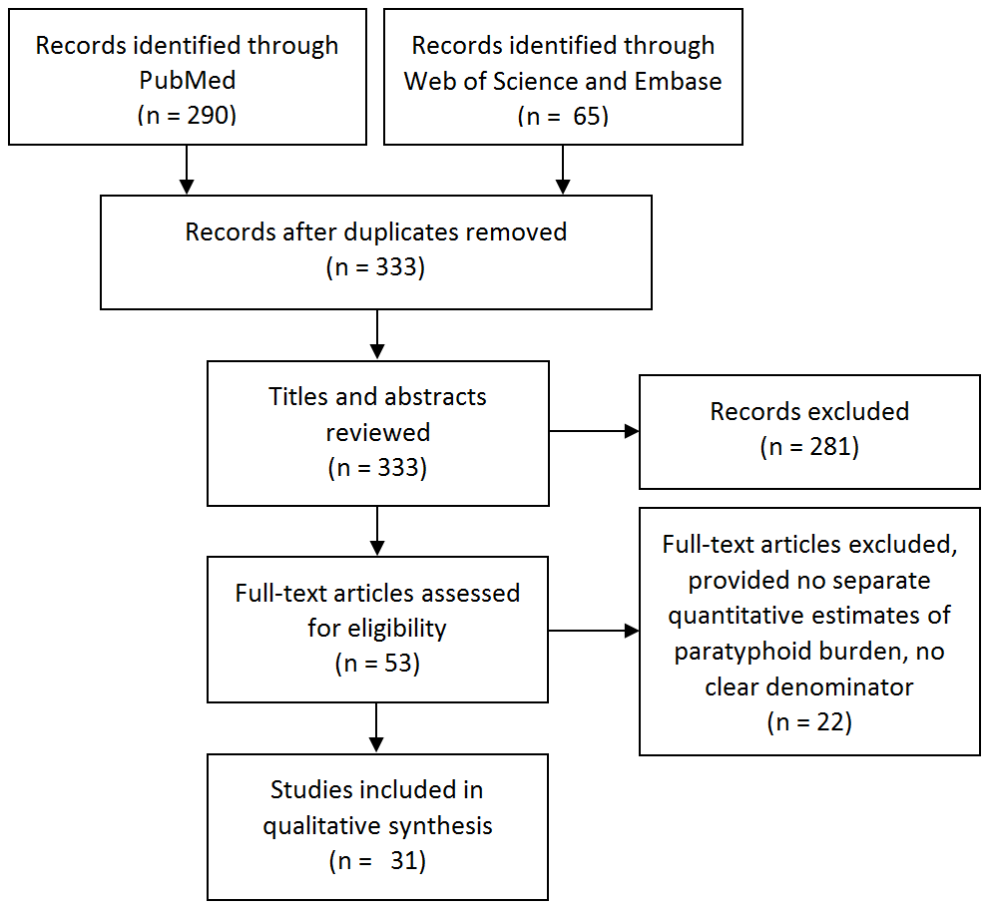
English-language systematic search results of *S.* Paratyphi A burden, articles published 2005–2013.

**Figure 3:**
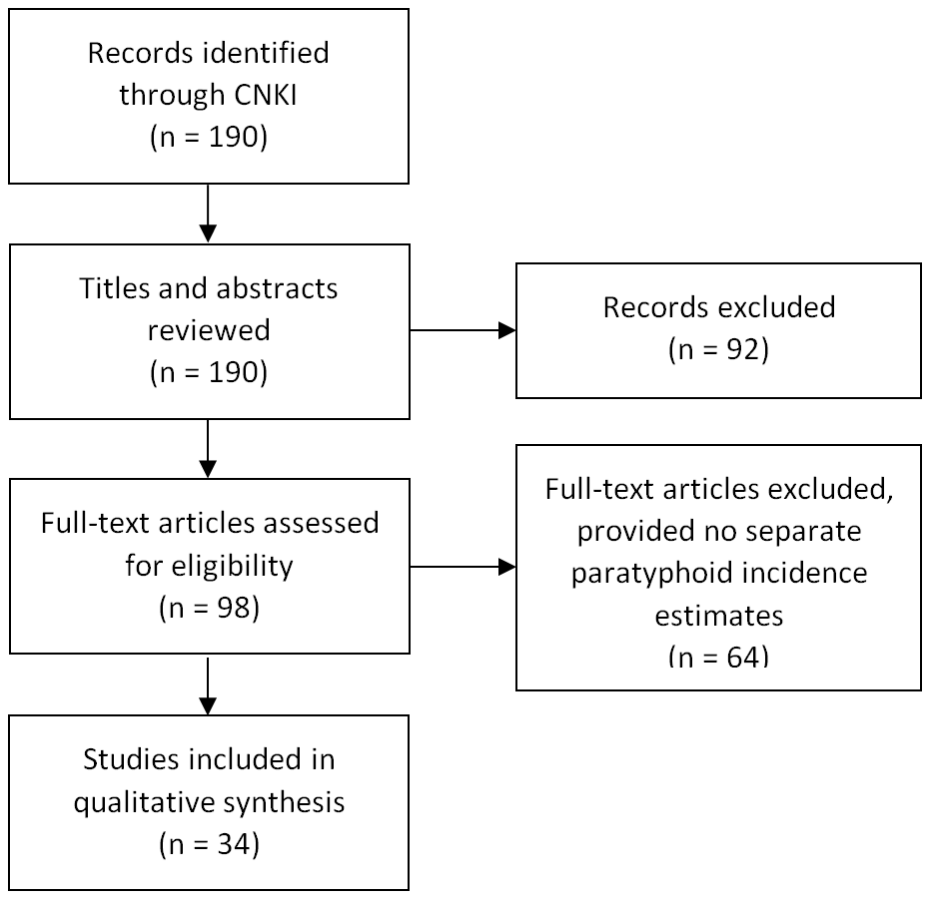
Chinese-language systematic search results of *S.* Paratyphi A burden, articles published 2005–2013.

We identified 523 titles for review: 333 titles from the English-language databases (Figure 2), and 190 titles from the Chinese-language database (Figure 3). Of the 333 titles in the English-language search, 31 full-text articles satisfied inclusion criteria and were included in the qualitative synthesis. A further 34 full-text articles were included in the qualitative synthesis from the Chinese-language database search. Tables 1 and 2 detail data extracted from English-language population-based and hospital-based studies, respectively, and table 3 details data extracted from Chinese-language population-based studies. We did not include diagnostic methods in these tables as there was very little heterogeneity across studies.

**Table 1:** Population-based surveillance studies which reported *S.* Paratyphi A burden in Asia and Africa.

Region	Reference	Year	Population	Sample size	Study design	Proportion of enteric fever *S.* Paratyphi A	Incidence (cases/100,000 person-years)	Quality score
East Asia								
Hongta District, China	[30]	2008–2009	District population	380,000	Passive surveillance	-	220 (est.)	Medium
Guangxi Province, China	[31]	2004	Province population	46,000,000	Passive surveillance	90.0% (est.)	10 (est.)	Medium
Hechi City, China	[12]	2001–2002	City population	3,830,000	Enhanced passive surveillance	64.3%	27	High
South Asia								
Karachi, Pakistan	[24]	2007–2008	Sample of children <5 years	5,570	Active surveillance	11.1%	51 (est.)	Medium
Dhaka, Bangladesh	[58]	2003–2004	Population sample	24,893	Active surveillance	16.7%	40	High
Kolkata, India	[11]	2003–2004	Population sample	56,946	Enhanced passive surveillance	37.0%	80	High
Kolkata, India	[12]	2003–2004	Population sample	57,075	Enhanced passive surveillance	23.5%	42	High
Karachi, Pakistan	[12]	8/2002–7/2003	Sample of 2–16 year olds	15,219	Passive surveillance	15.9%	76	High
Karachi, Pakistan	[23]	2002–2004	Sample of 2–16 year olds	116,500	Enhanced passive surveillance	14.5%	72	High
Southeast Asia								
Jakarta, Indonesia	[12]	2002–2003	Population sample	160,257	Enhanced passive surveillance	14.3%	13.7	High
Singapore	[28]	1990–2009	City population	5,183,700	Passive surveillance	45.4%	-	Low
Sub-Saharan Africa								
Lwak and Kibera Kenya	[6]	2007–2009	Individuals living in 0.4 km^2^ area (Kibera) and 100 km^2^ (Lwak)	28,000 (Kibera) 25,000 (Lwak)	Active surveillance	0%	0	High

**Table 2:** Hospital-based studies with proportional estimates of *S.* Paratyphi A burden in Asia and Africa.

Region	Reference	Year	Population (All ages unless noted)	Sample Size	Study design	Proportion of enteric fever *S.* Paratyphi A	Quality score
South Asia							
Kathmandu, Nepal	[14]	1993–2003	Suspected septicemia	82,467	Retrospective	29.3%	Medium
Kathmandu, Nepal	[16]	2002–2004	Patients with fever	103	Prospective	34.8%	Medium
Pokhara, Nepal	[26]	2000–2005	Pediatric cases of enteric fever	85	Retrospective case review	8.6%	Medium
Kathmandu, Nepal	[15]	6/2005–5/2009	Patients culture+ for Salmonella	3,898	Retrospective	64.6%	Medium
Kathmandu, Nepal	[25]	4/2007–3/2008	Febrile children <15	9,856	Prospective	17.0%	Medium
Kathmandu, Nepal	[17]	2008–2009	Culture-confirmed enteric fever	584	Case control study	26.2%	Medium
Kathmandu, Nepal	[59]	2008–2009	Patients with fever	479	Prospective	65.4%	Medium
Chandigarh, India	[21]	2003–2007	Patients with fever	11,240	Prospective (2006–7) and retrospective (2003–5)	24.3%	Medium
Mumbai, India	[19]	2003–2005	Patients with clinically diagnosed enteric fever	226	Retrospective chart review	38.7%	Medium
New Delhi, India	[27]	1999, 2002, 2005	Patients with fever	5,565	Prospective	10.3%	Medium
Rourkela, India	[20]	2005–2008	Patients with fever	5,340	Prospective	21.0%	Medium
South India	[18]	2005–2006	Patients with fever	6,010	Prospective	54.5%	Medium
Rawalpindi, Pakistan	[60]	1993–2003	Patients with fever	2,9104	Prospective	46.2%	Medium
Pakistan	[22]	2001–2006	Patients with fever	175,987	Prospective country-wide assessment	28.9%	Medium
Southeast Asia							
Phnom Penh, Cambodia	[29]	2007–2010	Adults with signs of Systemic Inflammatory Response Syndrome	6,881	Prospective	9.1%	Medium
Middle East							
Hamadan, Iran	[33]	2001–2004	Patients culture positive for Salmonella	296	Retrospective (banked specimens)	9.3%	Medium
Israel	[32]	1995–2003	Patients culture positive for S. Typhi, and *S.* Paratyphi A, B, an C	136	Retrospective country-wide assessment	13.4%	Medium
Sub-Saharan Africa							
Zaria, Nigeria	[61]	2007–2008	Patients with clinically diagnosed enteric fever	713	Prospective	17.4%	Medium
Lagos, Nigeria	[62]	2004–2005	Patients with fever or gastroenteritis	441	Prospective	34.4%	Medium
Dakar, Senegal	[9]	1996–2003	Patients with fever	10,944	Retrospective	0.0%	Medium
Benin	[7]	2005–2007	Patients with long-term fever, yellow diarrhea, and headache or dizziness	273	Prospective	2.3%	Low
Togo	[8]	2005–2006	Clinically diagnosed enteric fever	200	Prospective	0.0%	Medium

**Table 3:** Population-based studies which reported *S.* Paratyphi A burden in China.

Province	Reference	Sample size	Year	Population	Study design	Proportion of enteric fever *S.* Paratyphi A	Incidence (cases/100,000 person-years)	Quality score
Southwest China								
Yunnan	[41]	45,966,239	2005–2008	Province-based	Passive surveillance	35.1%	4.75	Medium
Yunnan	[63]	2,429,000	2005–2008	Baoshan City	Passive surveillance	21.3%	2.48	Medium
Guizhou	[64]	265,563	2006–2007	Liupanshui City	Active surveillance	83.3%	8.66	High
Guizhou	[65]	320,000	2001–2006	Ziyun County	Passive surveillance	59.1%	58.96	Medium
Guizhou	[66]	416,500 (2007)	2000–2007	Tianzhu County	Passive surveillance	2.0%	0.78	Medium
Guizhou	[42]	37,200,000	1999–2007	Province-based	Passive surveillance	26.5%	8.15	Medium
South China								
Guangxi	[67]	9,049	2001–2005	Sample from Hechi City	Active surveillance	70.7%	14.57	Medium
Guangxi	[68]	620,000	2002	Guilin City	Active surveillance	41.7%	0.02	Medium
Guangxi	[69]	13,379	2006–2007	Children from 38 primary, middle, and high schools	RCT/Active surveillance	-	185.00	High
Guangxi	[43]	47,680,000	2004–2010	Province-based	Passive surveillance	37.3%	4.78-0.8	Medium
Guangxi	[70]	120,097	2001–2005	Jinjiang	Active surveillance	73.1%	16.01	High
Guangxi	[71]	535,673	2008	Quanzhou County	Active surveillance	85.8%	180.00	High
Guangxi	[72]	4,930,000	1991–2005	Guilin	Passive surveillance	71.7%	32.64	Medium
Guangxi	[73]	6,480,000	2004–2007	Nanning	Passive surveillance	65.2%	0.82	Medium
Northeast China								
Liaoning	[34]	6,690,432	2006–2011	Dalian	Passive surveillance	20.6%	0.08	Low
East China								
Zhejiang	[37]	56,137,000	1994–2004	Province-based	Passive surveillance	67.6%	11.85	Medium
Zhejiang	[74]	525,900	2002–2008	Linan	Passive surveillance	98.7%	29.03	Medium
Zhejiang	[75]	46,260,000	2004–2006	Province-based	Active surveillance	92.0%	6.05	High
Zhejiang	[76]	1,500,000	2004	Taizhou	Passive surveillance	93.3%	57.45	Medium
Zhejiang	[77]	402,300	2001–2004	Yuhuan County	Passive surveillance	99.8%	192.50	Medium
Zhejiang	[78]	1,150,000	2004	Wenling	Passive surveillance	100.0%	163.26	Medium
Zhejiang	[79]	583,000	2004–2007	Jiangshan City	Passive surveillance	74.6%	4.89	Medium
Zhejiang	[80]	7,600,000	2004–2006	Ningbo	Passive surveillance	-	29.58	Medium
Zhejiang	[81]	691,900	2004–2008	Zhoushan	Passive surveillance	66.2%	3.90	Low
Zhejiang	[82]	1,018,000	2000–2006	Shaoxing	Passive surveillance	70.3%	13.88	Medium
Zhejiang	[38]	46,260,900	1997–2006	Province-based	Passive surveillance	79.9%	8.61	Medium
Shandong	[39]	92,480,000	2005	Province-based	Passive surveillance	69.7%	0.19	Medium
Shandong	[83]	11,000,000	2005	Linyi	Passive surveillance	98.7%	5.32	Low
			2006			96.7%	1.32	
			2007			97.1%	2.36	
Shandong	[84]	9,942,000	2004	Linyi	Passive surveillance	98.7%	5.23	Medium
			2005			96.7%	1.43	
Fujian	[40]	37,106,188 (2010)	2004–2010	Province-based	Passive surveillance	25.4%	0.34	Medium
South central China								
Hunan	[44]	2,490,275	2003–2010	Xiangxi	Passive surveillance	10.5%	0.57	Medium
Hunan	[45]	548,000	2001–2010	Lengshuitan	Passive surveillance, active surveillance since 2004	6.6%	0.91	Medium
Central China								
Henan	[85]	668,592	2007	Dengfeng City	Passive surveillance		25.98	Low
Henan	[36]	93,880,000	2011	Province-based	Passive surveillance	36.0%	0.06	Medium

### Paratyphoid A in Africa

We identified a single population-based study which provided an estimate of *S.* Paratyphi A incidence in Africa (Table 1) and five hospital-based studies from African countries with estimates of the proportion of enteric fever due to *S.* Paratyphi A (Table 2). The hospital-based studies were conducted in the West African countries of Nigeria, Senegal, Togo, and Benin. None of the studies were considered nationally representative.

The single population-based study was conducted in Kenya and utilized active surveillance for enteric fever in a population of 57,000 living in an informal settlement and rural area [6]. Typhoid fever incidence was estimated as 247 cases/100,000 person-years but no cases of *S.* Paratyphi A were detected.

In the five African hospital-based studies, the proportion of enteric fever cases attributable to *S.* Paratyphi A ranged from 0 to 34.4%. The two prospective studies conducted in Nigeria found that 17.4% of enteric fever cases in Zaria, Nigeria between 2007 and 2008 and 34.4% of enteric fever cases in Lagos, Nigeria between 2004 and 2005 were due to *S.* Paratyphi A. The other studies from West Africa identified between 0 and 2% of enteric fever isolates as *S.* Paratyphi A [7]–[9].

### Paratyphoid A in Asia

#### South Asia

We identified six population-based studies with *S.* Paratyphi A incidence estimates in South Asia (Table 1) and fourteen hospital-based studies with estimates of the proportion of enteric fever due to *S.* Paratyphi A (Table 2). These studies were conducted in regions of Nepal, India, Pakistan, and Bangladesh. Only one of the hospital-based studies was considered to be nationally representative. The population-based studies used enhanced passive or active surveillance methods. Five of the six the population-based studies in South Asia were of high quality.

*S.* Paratyphi A incidence estimates from South Asian countries ranged from 40 to 80 cases per 100,000 person-years in the general population. The lowest *S.* Paratyphi A incidence estimate came from an urban sample in Dhaka, Bangladesh between 2003 and 2004 [10]. The highest paratyphoid incidence was observed in an urban sample from Kolkata, India between 2003 and 2004 [11], [12]. Population-based studies in South Asia reported that 14.5–37% of enteric fever cases were attributable to *S.* Paratyphi A while hospital-based studies estimated 10.3–54.5% of enteric fever cases attributable to *S.* Paratyphi A. There appeared to be significant regional variability in the estimates of *S.* Paratyphi A incidence in South Asia.

Several hospital-based studies including patients of all ages were identified from Nepal, where *S.* Paratyphi A was identified in between 26 and 65% of culture-positive enteric fever cases [13]–[17]. Hospital-based studies from India demonstrated great regional variation in proportion of enteric fever attributed to *S.* Paratyphi A as 38.7 and 54.5% of enteric fever isolates were identified as *S.* Paratyphi A in South and West India respectively compared to only 21.0 and 24.3% in East and North India respectively [18]–[21]. The proportion of *S.* Paratyphi A in Pakistan was similar to East and North India, where a prospective country-wide hospital-based study identified 28.9% of enteric fever isolates as *S.* Paratyphi A between 2001 and 2006 [22].

Four studies from South Asia focused specifically on pediatric populations. The incidence estimates reported from pediatric population-based studies (51–76 cases/100,000 person-years) did not differ substantially from incidence reported in adults and children from South Asian countries [23], [24]. In all pediatric studies, *S.* Paratyphi A accounted for a lower proportion of enteric fever cases (8.6–17.0%) than in studies which included adults, with the exception of one study conducted in New Delhi, India [23]–[27].

#### Southeast Asia

We identified one hospital-based and two population-based studies from Southeast Asia. These studies were conducted in Indonesia, Cambodia, and Singapore. The study conducted in Singapore was considered to be nationally representative.

A high quality, enhanced passive surveillance study in Jakarta, Indonesia estimated that the *S.* Paratyphi A incidence rate was 14 cases/100,000 person-years between 2002 and 2003, and the proportion of enteric fever due to *S.* Paratyphi A was 13.7% [12]. A passive surveillance study conducted in Singapore did not provide an incidence estimate, but estimated that 45.4% of “indigenous” enteric fever cases between 1990 and 2009 were *S.* Paratyphi A [28]. This estimate excluded imported cases of enteric fever, as prior data suggested a high rate of imported paratyphoid cases. More than half the cases occurred during an epidemic in 1996.

The one hospital-based study from Southeast Asia included adult patients above the age of 8 and was conducted in Phnom Penh, Cambodia, where *S.* Paratyphi A was identified in 9.1% of culture-positive enteric fever cases [29].

#### East Asia

We identified three population-based studies with *S.* Paratyphi A incidence estimates in East Asia from the English-language search, and no hospital-based studies. All of these studies were conducted in regions of China, and none were considered nationally representative. The studies used passive or enhanced passive surveillance methods, and estimates ranged from 10 to 220 cases per 100,000 person-years.

The highest quality study utilized enhanced passive surveillance and estimated an *S.* Paratyphi A incidence of 27 cases/100,000 person-years in Hechi City (Guangxi Province) between 2001 and 2002, with *S.* Paratyphi A accounting for 64.3% of enteric fever cases [12]. The other two studies used passive surveillance methods in mixed urban and rural settings [30], [31]. The estimated incidence of *S.* Paratyphi A was 10 cases/100,000 person-years in Guangxi Province in 2004, with an estimated 90% of enteric fever attributable to *S.* Paratyphi A [31]. The incidence of *S.* Paratyphi A was 220 cases/100,000 person-years in Hongta District (Yunnan Province) between 2008 and 2009 [30].

#### Middle East

Very little data are available from countries in the Middle East. We identified two hospital-based studies from countries in the Middle East which provided estimates of the proportion of enteric fever due to *S.* Paratyphi A. These studies were conducted in Israel and Iran, and the study from Israel was considered to be nationally representative.

A retrospective country-wide assessment of enteric fever cases in Israel estimated that 13.4% of enteric fever cases between 1995 and 2003 was caused by *S.* Paratyphi A [32]. A cross-sectional study in Hamadan, Iran examined banked specimens collected from 2001–2004 and estimated that 9.3% of enteric fever cases were attributable to *S.* Paratyphi A [33].

### Paratyphoid A in China (Chinese-language studies)

From the Chinese-language search of the CNKI, we identified a total of 34 articles reporting on population-based studies with incidence estimates for *S.* Paratyphi A, conducted in Southwest, South, Northeast, East, Central South, and Central regions of China (Table 3). Most of these studies also estimated the proportion of enteric fever due to *S.* Paratyphi A. While none of the studies were nationally representative, nine articles reported province-level estimates, which pertain to populations of several million people. The majority of the studies utilized passive surveillance methods. Included studies were mostly medium or high quality.

*S.* Paratyphi A incidence estimates in China ranged from 0.08 to 192.5 cases per 100,000 person-years. The proportion of enteric fever cases attributable to *S.* Paratyphi A also ranged widely: from 0.6 to 98.7%. The lowest incidence estimate came from Dalian City in the Northeast region (Liaoning Province) between 2006 and 2011 [34]. The highest paratyphoid incidence was from the Zhejiang Province in the East region of China between 2001 and 2004 [35]. There were a wide range of incidence rates observed in the 11 studies conducted in the region between 1994 and 2008.

Proportion of enteric fever due to *S.* Paratyphi A was geospatially clustered. The highest proportional estimates were in the East region, 70 and 99% of isolates identified between 1994 and 2008 in Zhejiang Province attributable to *S.* Paratyphi A during this time. These estimates are similar to reports from neighboring Shandong Province between 2004 and 2007 (67–99%).

Province-level estimates using passive surveillance methods ranged from 0.06 to 11.85 cases of *S.* Paratyphi A per 100,000 person-years. In the Central region, the Henan Province had the lowest incidence estimate in 2011 and Zhejiang Province in the East region had the highest incidence between 1994 and 2004 [36], [37]. However, incidence between 1997–2005 in Zhejiang Province was 8.61 cases/100,000 person-years [38]. Shandong and Fujian Provinces in the East region had *S.* Paratyphi A incidences of 0.19 (2005) and 0.34 (2004–2010) cases per 100,000 person-years, respectively [39], [40]. In the Southwest region, the *S.* Paratyphi A incidence rate was 4.75 and 8.15 cases per 100,000 person-years, in Yunnan (2005–2008) and Guizhou (1999–2007) Provinces respectively [41], [42]. In the South region, the incidence of *S.* Paratyphi A in Guangxi Province dropped from 4.78 to 0.8 cases per 100,000 person-years between 2004 and 2010 [43]. In the Central South region, Hunan Province had an estimated *S.* Paratyphi A incidence of between 0.57 and 0.91 cases per 100,000 person-years between 2001 and 2010 [44], [45].

## Discussion

Global estimates of the burden of paratyphoid as a cause of enteric fever are limited by a lack of systematic data collection and surveillance. However, available data suggest that when attempts have been made to identify *Salmonella* species in blood culture isolates, *S.* Paratyphi A is almost always identified and often constitutes a significant proportion of all cases of enteric fever, particularly in Asia. This paper focuses on regional estimates of *S.* Paratyphi A within Africa and Asia. In addition to an English-language search, we included a thorough review of Chinese-language databases and identified a significant amount of data available from China. Unlike previous reviews of the subject, we have also presented two separate measures of burden; both incidence and proportion of enteric fever attributable to paratyphoid.

Overall, we found a high burden of paratyphoid fever across countries in Asia, both in terms of incidence and in the proportion of enteric fever attributable to *S.* Paratyphi A. The highest incidence estimates came from China, where four studies estimated incidence rates of over 150 cases/100,000 person-years. The largest incidence estimate came from Hongta District in China; where in 2002 there were 220 cases/100,000 person-years reported. The highest proportional estimate from Asia also came from China (Yuhuan County), where between 2000 and 2004, an estimated 99.8% of enteric fever cases were attributable to paratyphoid. However, estimates of paratyphoid burden in Asia were not consistently high; for example, nearly a third of the Chinese studies reported incidence estimates of less than 1 case/100,000 person-years. The proportion of enteric fever cases attributable to paratyphoid from population and hospital-based studies in Asia was also highly variable, with reported estimates ranging from 2 to 99.8%.

Data from Africa estimating the burden of paratyphoid A are extremely limited. The single population-based study from Africa (Kenya) which reported separate estimates of *S.* Paratyphi A and *S.* Typhi did not identify any cases of paratyphoid and hospital-based studies from Senegal, Benin, and Togo reported that less than 2% of enteric fever cases were attributable to *S.* Paratyphi A [6]–[9]. However, two hospital-based studies in Nigeria found that a considerable proportion of enteric fever cases (17–34%) were attributable to paratyphoid A [46], [47]. Although these studies suggest that Africa may have a lower incidence of *S.* Paratyphi A than Asia, the small number of studies available and the limited populations sampled may have underestimated the true burden of disease on the continent.

There are a number of potential explanations for the wide regional variation in reported paratyphoid A burden. There was considerable variability in the time period reported across studies, with some studies reporting data for several year periods and others reporting data from discrete seasons or shorter periods of time. Given known fluctuations in enteric disease incidence and pathogen-specific attributable risk during high-precipitation months, oversampling during these periods may have introduced bias into the incidence estimates from some population-based studies [48], [49]. In addition, laboratory practices may vary by setting and the majority of studies detected *S. enterica* by blood culture (with serovar identification by O and H serology) which has low sensitivity in comparison with less-commonly performed bone-marrow culture [50], [51]. Assuming that blood culture is equally sensitive to *S.* Typhi and *S.* Paratyphi, it is likely that the population-based incidence estimates are underestimates of the true burden. The substantial variation in *S.* Typhi vaccine coverage (which has been available for over twenty years in some areas) may also have affected proportional estimates of paratyphoid burden. Finally, care-seeking and treatment patterns differ across geographies and populations and in areas of low care-seeking and treatment. Therefore the reported burden of paratyphoid may have been underestimated in many of the included studies.

This review highlights the critical need to expand surveillance activities to define the burden of paratyphoid throughout Asia and Africa, where enteric fever is a relatively common cause of morbidity and mortality. The large number of population-based studies from China demonstrates the utility of an extensive communicable disease surveillance system. The Chinese National Infections Disease Surveillance System (NIDSS) mandates that all hospitals and village clinics report both suspected and confirmed cases of paratyphoid and typhoid within 24 hours using an internet-based real-time case reporting system [52]. Expanding global surveillance using similar platforms would improve policymakers' ability to allocate appropriate resources to the regions where they are needed most. There are many underlying reasons for the absence of such surveillance systems in Africa and much of Asia. One of the main barriers is that a confirmed diagnosis of enteric fever requires blood or bone-marrow culture, and most clinical facilities in these regions lack such capacity, due in great part to limited human and financial resources. The limited capacity leads to missed diagnoses and inappropriate treatment for many severe infections, and hinders the estimation of global burden of *S.* Typhi, *S.* Paratyphi A, and other invasive and enteric pathogens. Therefore the improvement of laboratory capacity in developing settings is an important goal which would facilitate public health progress and clinical services. Additionally, we should encourage the development of highly sensitive and specific point-of-use diagnostics for enteric fever and other pathogens which have the potential to immediately reduce disease morbidity and mortality.

While the increased coverage of *S.* Typhi Vi polysaccharide and Ty21a vaccine in many regions has reduced typhoid fever incidence, there is only limited immunological evidence of the Ty21a vaccine's cross protection for *S.* Paratyphi A [53], [54]. However, a study conducted in Santiago, Chile reported that Ty21a had 49% efficacy for *S.* Paratyphi B [55]. The high incidence and proportion of paratyphoid A in some areas suggests the possible utility of an *S.* Paratyphi A vaccine. Concerns about “serovar replacement” were raised after surveillance in Guangxi China suggested an increase in *S.* Paratyphi A incidence, coincident with a decrease in *S.* Typhi cases, as Vi polysaccharide vaccination coverage increased [31]. However, in the subsequent 6 years, the incidence of *S.* Paratyphi A decreased to mirror that of *S.* Typhi [43]. Increases in the proportion of enteric fever attributable to *S.* Paratyphi A were observed in hospitals in Bangkok, Thailand in the early 1980s following the commencement of a major typhoid vaccination campaign among schoolchildren in 1977 [56]. As the study utilized an early form of typhoid vaccine and was conducted amidst a typhoid epidemic which occurred from 1974–1979, it was difficult to draw conclusions regarding serovar displacement. While we were unable to assess serovar replacement in this systematic analysis due to the lack of repeat estimates from the same region, the potential emergence of paratyphoid as a common cause of enteric fever in areas with high *S.* Typhi vaccine coverage further supports the importance of increased global surveillance.

This review included studies from a comprehensive search of both English and Chinese literature. In addition, the systematic search methods employed and the focus on both Africa and Asia add considerably to the available literature. However, this analysis also has several significant limitations. The majority of paratyphoid burden data from Asia and Africa came from hospital-based studies, which may not be representative of the general population. In addition, most of the population-based studies were from urban rather than rural or mixed settings, which may overestimate incidence estimates relative to the general population. It is important to note that the paucity of population-based studies from Africa which report *S.* Paratyphi A incidence does not mean that bacteremia surveillance studies have not detected paratyphoid. Bacteremia and fever surveillance have been conducted in several regions of Africa through the HiB initiative and malaria vaccine research respectively. Publications from these studies have focused on target organisms, have either not reported enteric fever incidence or not provided separate estimates for *S.* Paratyphi A and *S.* Typhi. Therefore such studies were not identified in this review. The search for literature on paratyphoid burden was conducted using a specific set of search terms, in order to target the most relevant articles. However it is likely that a small number of relevant articles meeting our inclusion criteria were not produced using these terms, such as a hospital-based pediatric study conducted in Cambodia which reported that 2% of enteric fever was attributable to *S.* Paratyphi A [57]. Finally, most of the population-based studies utilized passive surveillance, which may under-ascertain cases and may underestimate the true burden of paratyphoid in those regions.

The Global Burden of Diseases Study (GBD 2010) demonstrated a large confidence interval for the number of deaths worldwide due to enteric fever. It is clear that death due to enteric fever is not being recorded properly and is poorly captured by verbal autopsy studies that are the major source of cause of death data in many developing countries. The availability of a vaccine for *S.* Typhi makes differentiating typhoid and paratyphoid A burden crucial for an enteric fever control program and efficient resource allocation. This analysis demonstrates significant heterogeneity in the proportion of enteric fever due to *S.* Typhi and highlights the public health challenges facing control efforts, including the potential for serovar replacement following typhoid vaccination.

The high variability in documented incidence and proportion estimates of paratyphoid A suggest considerable geospatial variability in the burden of paratyphoid fever. We identified significant gaps in existing population-level estimates of paratyphoid A burden in Asia and Africa, as the majority of data comes from hospital-based studies. However, available data suggest that paratyphoid A is a significant cause of enteric fever in Asia. There are very little data from hospital-based or population-based studies in Africa, although proportional estimates of paratyphoid burden from Nigeria suggest that *S.* Paratyphi A may be common in Africa as well. China's NIDSS provides a good model for surveillance, and other populations in Asia and in Africa would greatly benefit from the creation and use of similar data collection platforms. Additional efforts to monitor enteric fever at the population level will be necessary in order to accurately quantify the public health threat posed by *S.* Paratyphi A, and to improve the coordination of prevention activities.

## Supporting Information

Checklist S1 PRISMA checklist for *Estimating the Burden of Paratyphoid A in Asia and Africa*.
(DOCX)
